# Supplementary material for: Structure and mechanism of the RNA dependent RNase Cas13a from Rhodobacter capsulatus
Source: Commun Biol. 2022 Jan 20;5:71. doi: 10.1038/s42003-022-03025-4 (PMC8776769; doi:10.1038/s42003-022-03025-4)
Supplement: Supplementary file 2 — Supplementary Information [file 42003_2022_3025_MOESM2_ESM.pdf]

Supplementary information

Structural and mechanistic insights into Cas13a from *Rhodobacter capsulatus*

Kick, L.M.,[1] von Wrisberg,[1] M.-K., Runtsch, L. S.[1] and Schneider, S.*[1]

[1]Ludwig-Maximilians University, Department of Chemistry, Institute for Chemical Epigenetics, Würmtalstr. 201, 81375 Munich Germany

E-mail: sabine.schneider@cup.lmu.de

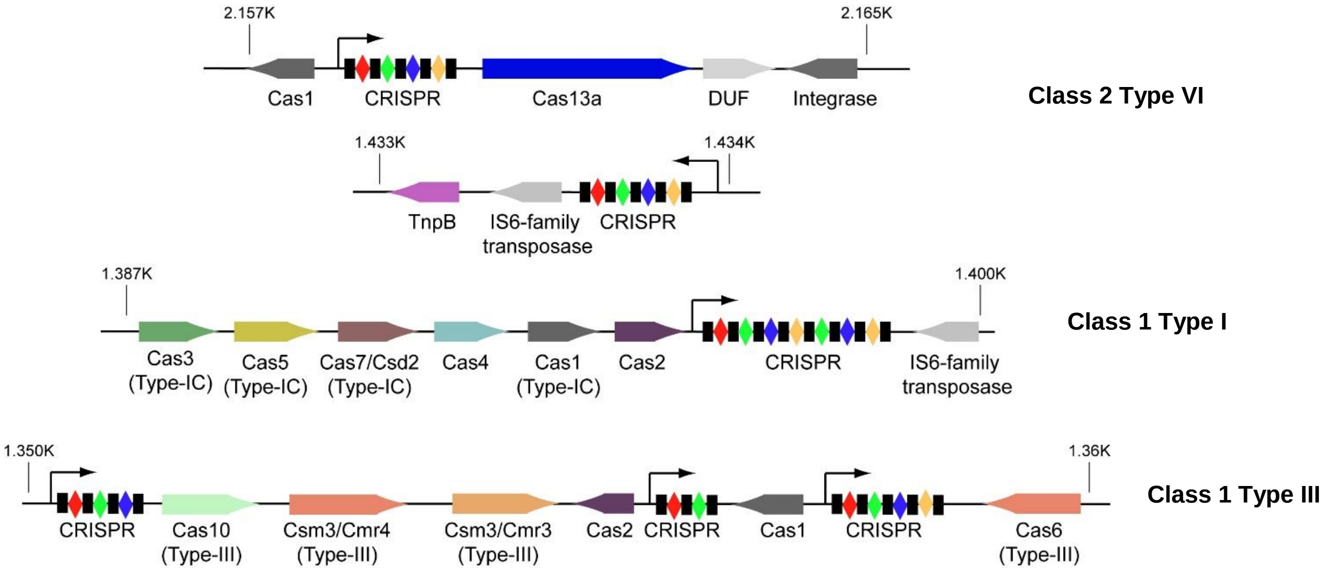

Supplementary Figure S1. Genomic location and structure of CRISPR-Cas sequences identified in the reference genome of *Rhodobacter capsulatus SB1003* (NCBI accession number NC_014034.1) using the CRISPR-finder(1) and BLAST search(2).

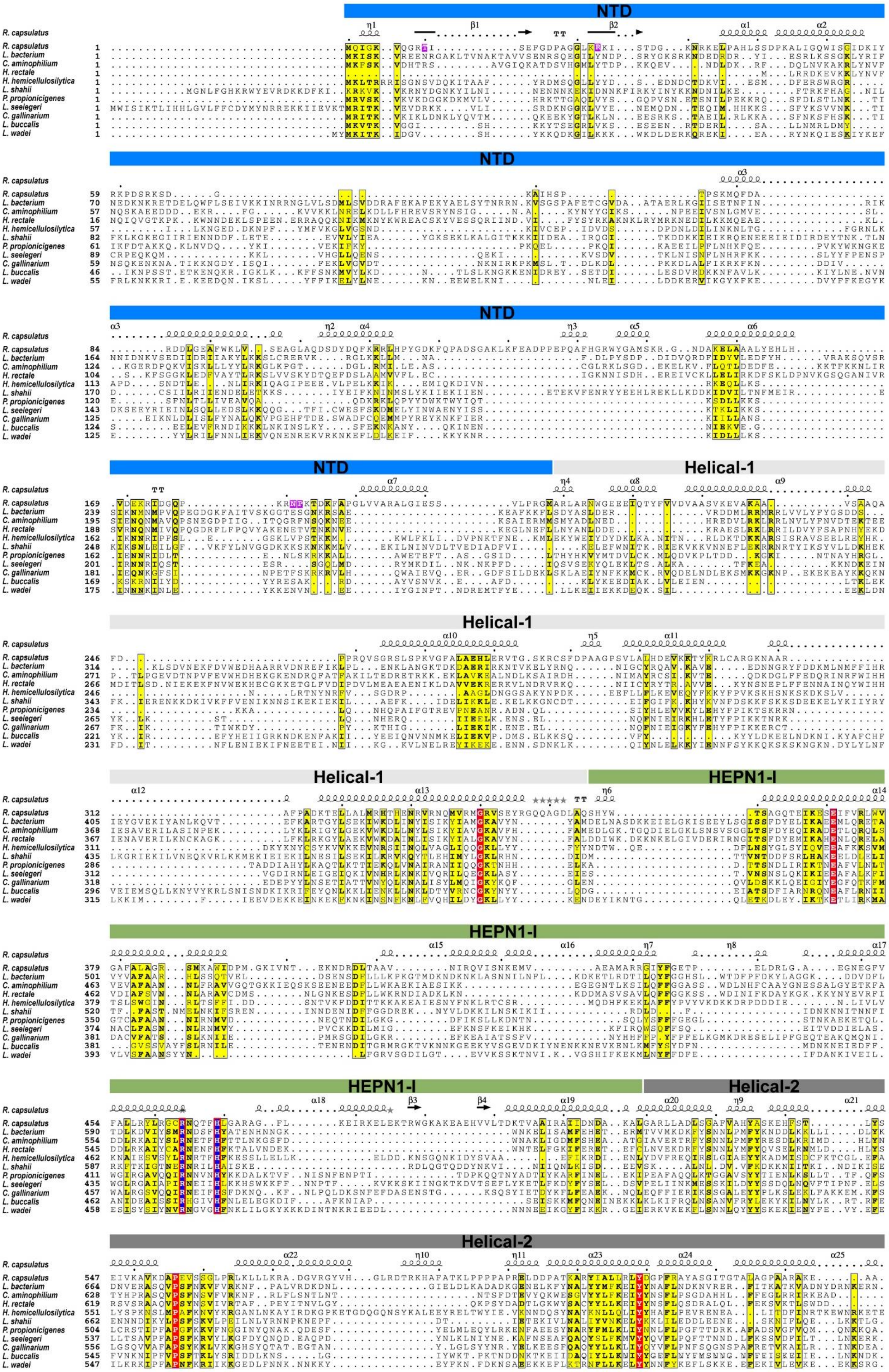
NTD
Helical-1
HEPN1-I
Helical-2
R. capsulatus
L. bacterium
C. aminophilium
H. rectale
H. hemicellulosilytica
L. shahii
P. propionicigenes
L. seelegeri
C. gallinarium
L. buccalis
L. wadei

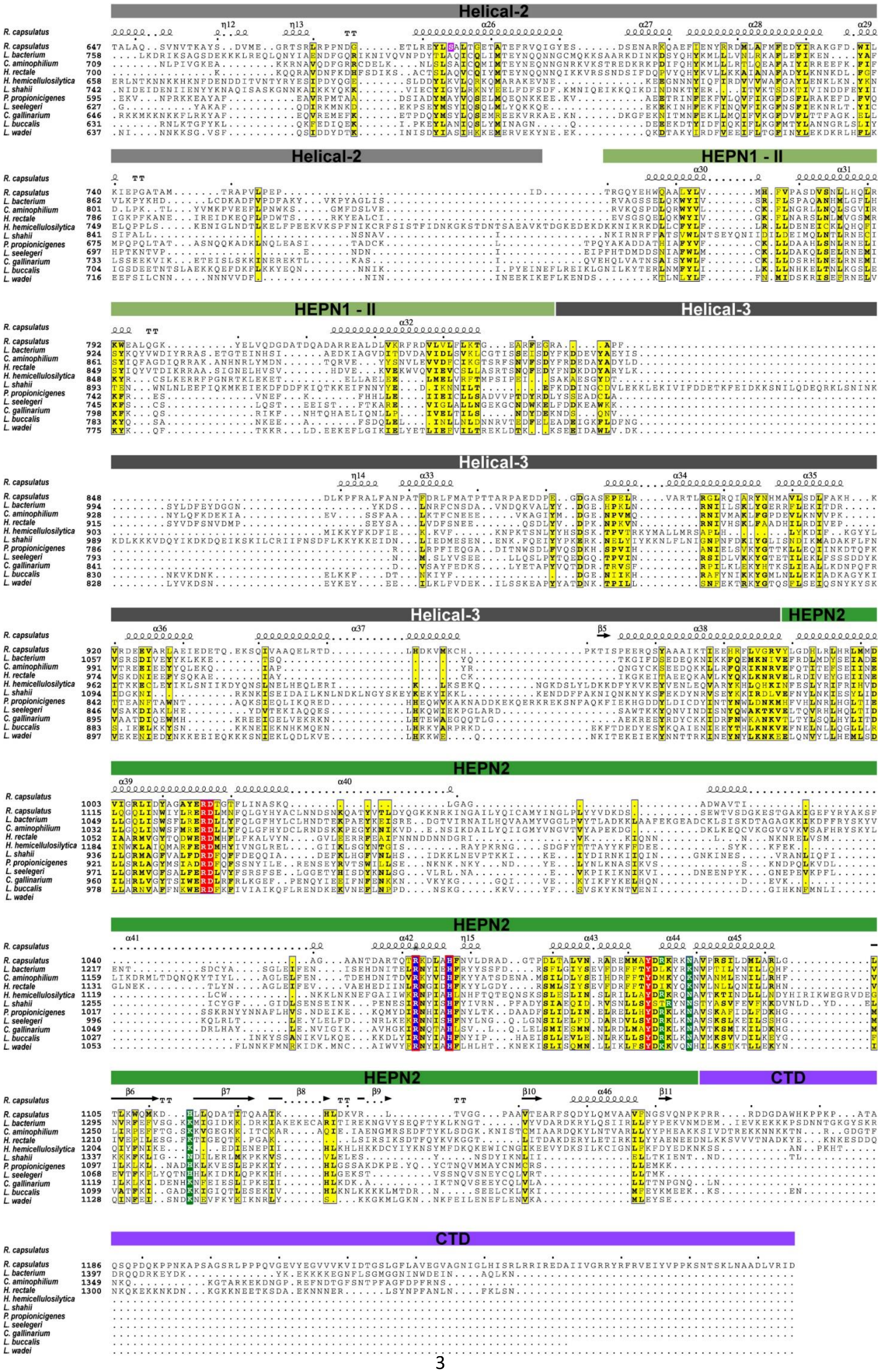
Helical-2
HEPN1 - II
Helical-3
HEPN2
CTD
R. capsulatus
L. bacterium
C. aminophilium
H. rectale
H. hemicellulosilytica
L. shahii
P. propionicigenes
L. seelegeri
C. gallinarium
L. buccalis
L. wadei

Supplementary Figure S2: Sequence alignment of Cas13a homologs. Secondary structure assignments according to the RcCas13a-crRNA binary complex structure presented in this work. Catalytic residues of the HEPN domains and residues responsible for pre-crRNA processing are highlighted in blue and green, respectively. Amino acids forming sequence-specific contacts with the crRNA are shown in pink. Conserved aromatic and aliphatic regions are shaded yellow, charged residues in red. NCBI RefSeq / Uniprot ID: *R. capsulatus*: WP_013067728.1 / D5AUW0, *L. bacterium*: WP_022785443.1 / P0DPB7, *C. aminophilum*: WP_031473346.1, *E. rectale*: WP_055061018.1, *H. hemicellulosilytica*: WP_103203632.1 / A0A0H5SJ89, *L. shahii*: WP_018451595.1 / P0DOC6, *P. propionicigenes*: WP_013443710.1 / E4T0I2, *L. seeligeri*: WP_012985477.1 / P0DPB8, *C. gallinarum*: WP_034560163.1, *L. buccalis*: WP_015770004.1 / C7NBY4, *L. wadei*: WP_021746003.1 / U2PSH1. Alignment was performed with ClustalX (3) and annotated manually and using ESPript (4).

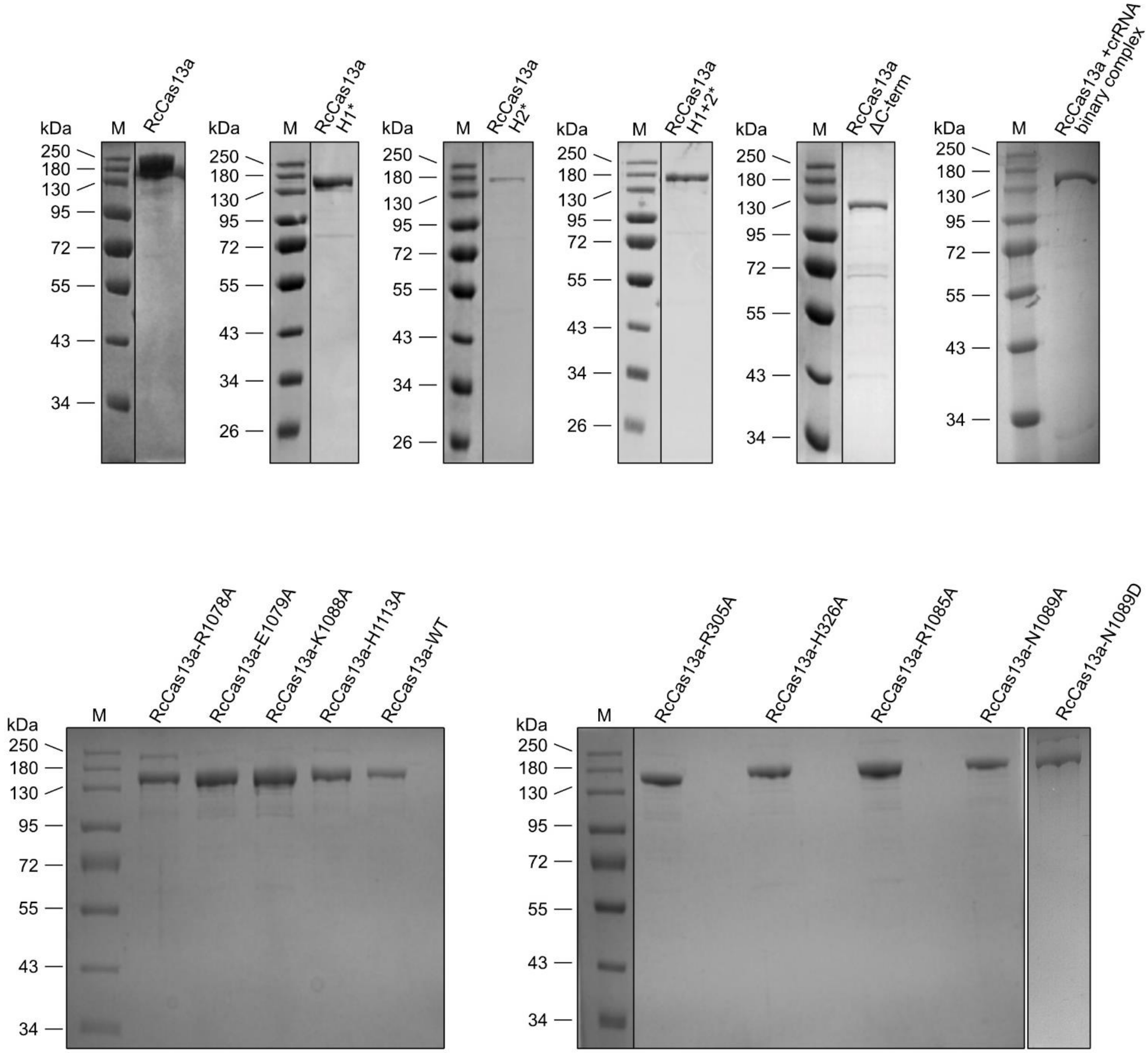

Supplementary Figure S3. SDS-PAGE analysis (Comassie blue staining) of purified wild type RcCas13a, RcCas13a mutants and the RcCas13a-crRNA complex. RcCas13a$^{R464A/H469A}$; H2*=RcCas13a$^{R464A/H469A}$; H1+2*=RcCas13a$^{R464A/H469A/R1052A/H1057A}$; ΔC-term = Δ C-terminal domain.

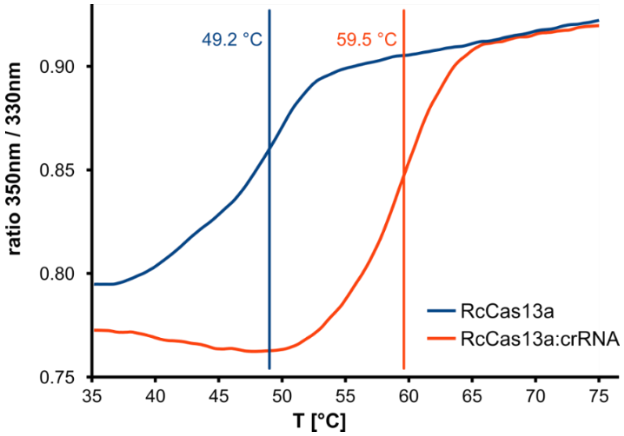

Supplementary Figure S4: Thermal stability of RcCas13a and the RcCas13a binary complex. Melting curves of apo-RcCas13a (blue) and RcCas13a-crRNA binary complex (orange) determined by nanoDSF (Nanotemper Tycho NT.6) using standard settings.

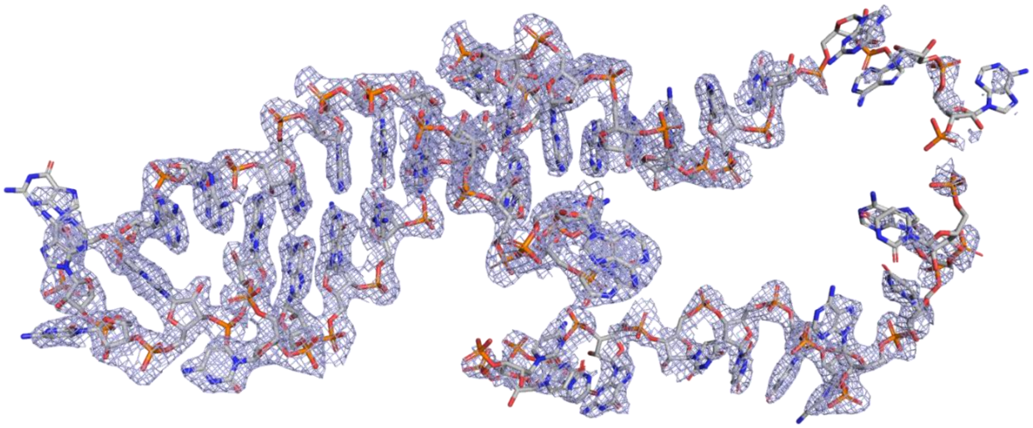

Supplementary Figure S5: Simulated annealing omit Fo-*D*Fc map of the crRNA chain, contoured at 2.5σ calculated with PHENIX. Parts of the spacer region are flexible and not well defined in the electron density.

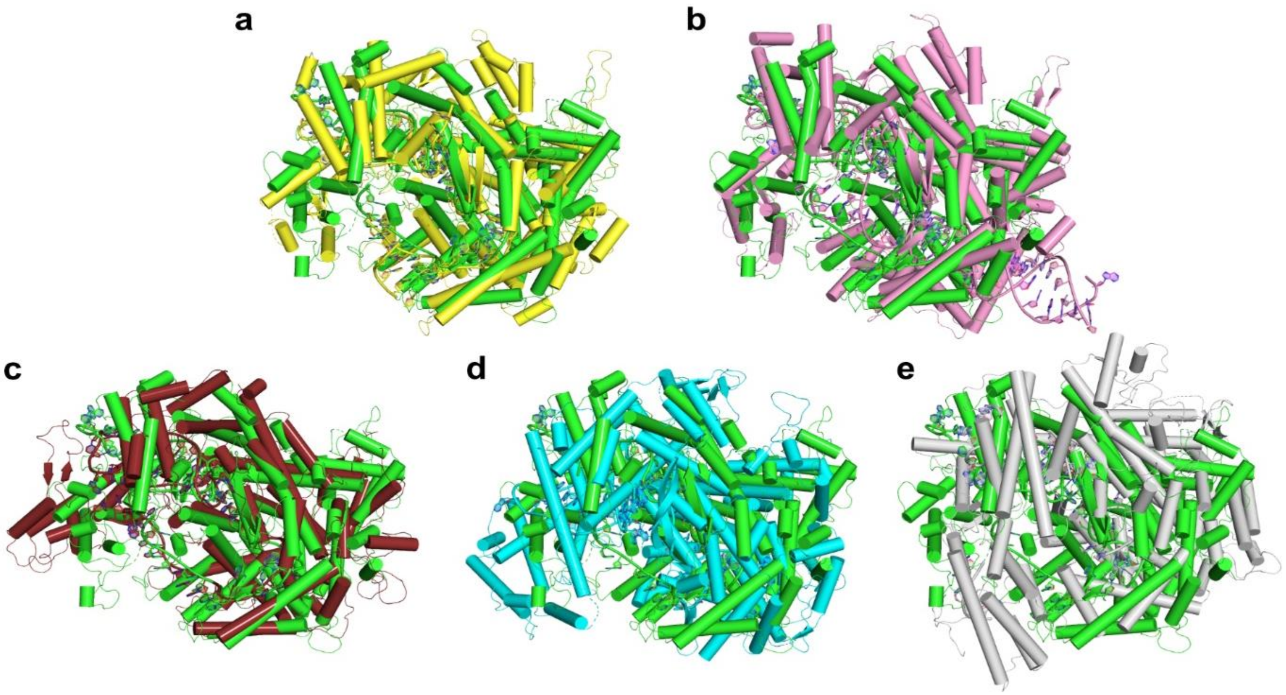

Supplementary Figure S6. Superposition of RcCas13a with homologous structures. RcCas13a-crRNA binary complex (green) with a) LbuCas13a-crRNA binary complex (yellow, PDB code 5XWY) b) LbuCas13a-crRNA-targetRNA ternary complex (pink, PDB code 5XWP), c) LseCas13a-crRNA binary complex (brown, PDB code 6VRC), d) LbaCas13a-crRNA binary complex (cyan, PDB code 5W1I), e) LshCas13a-crRNA binary complex (gray, PDB code 5WTK).

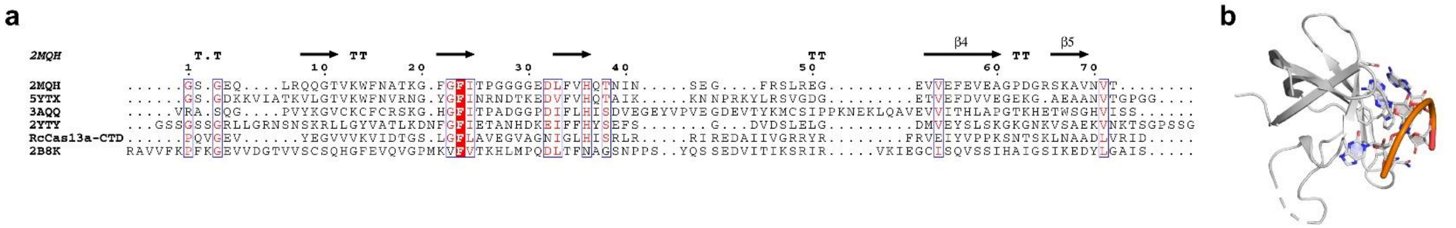

Supplementary Figure S7. a) Sequence alignment of the RcCas13a-CTD and homologs identified with HHPRED,(5) annotated using ESPript.(4) b) Structure of the human Y-box binding protein 1 (YB-1), a member of the cold shock domain (CSD) protein family, in complex with RNA (PDB code 5YTX). RcCas13a might adopt an overall similar folding topology.

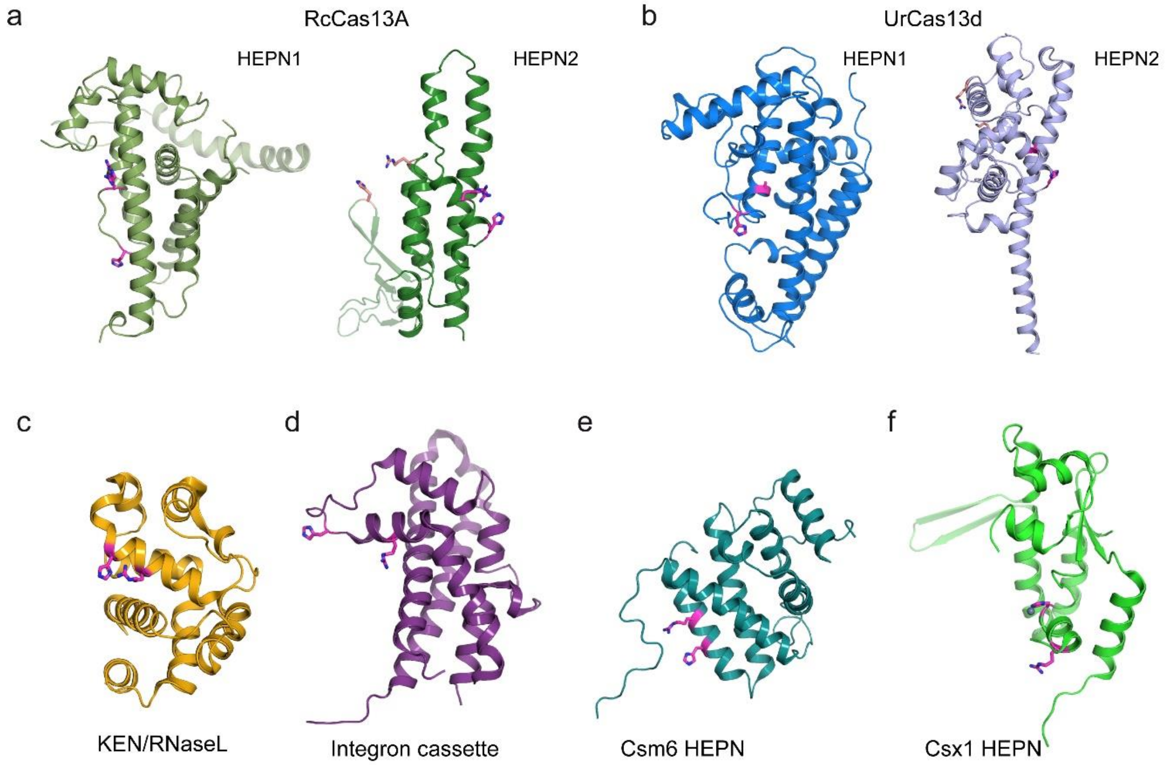

Supplementary Figure S8. Cartoon representation of HEPN-domains highlighting their common helical folding topology and diversity. a) HEPN1 and HEPN2-domains of RcCas13a. b) uncultured *Ruminococcus sp. C*as13d (PDB code 6IV9) c) *Saccharomyces cerevisiae* RNaseL (PDB code 3LJ2), d) *Vibrio paracholerae* Integron cassette protein (PDB code 3JRT), e) HEPN-domain of *Thermus thermophiles* Csm6 (PDB code 5FSH), f) HEPN-domain of *Pyrococcus furiosus* Csx1 (PDB code 4EOG). The R(x)$_{4-6}$H-signature motif is highlighted as pink stick model, residues involved in pre-crRNA by RcCas13a and UrCas13d are shown in light pink.

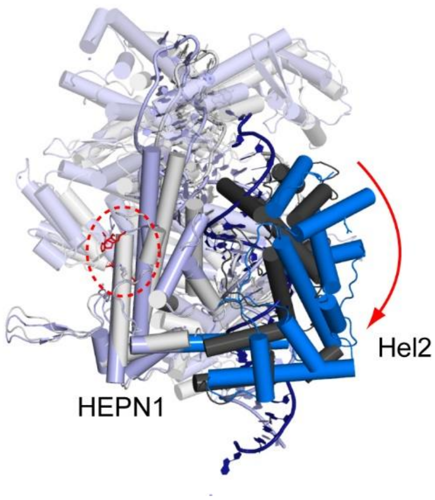

Supplementary Figure S9. Superposition of the binary (PDB code 5XWY, grey) and ternary complex structures (PDB code 5XWP, blue) of LbuCas13a. Upon target RNA (dark blue) binding the Hel 2 domain rotates about 25° relative to the HEPN1 domain, resulting in the rearrangement of the catalytic residues in the active site (red circle).

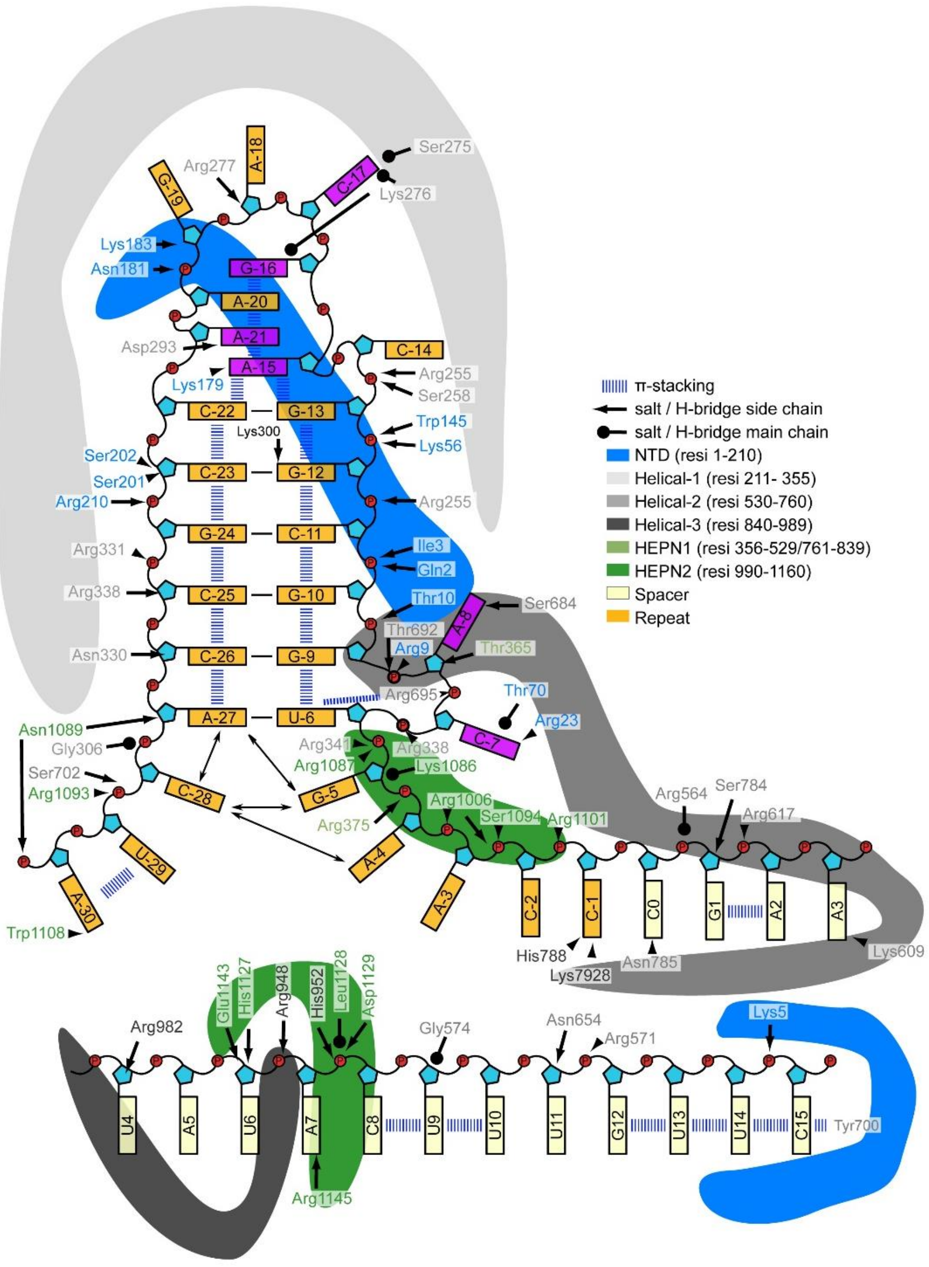

Supplementary Figure S10: Scheme showing the interactions between crRNA and RcCas13a. Residues from different protein domains are color coded. Base-specific contacts only occur to the stem-loop and 3' flank. Only 16 nt's of the spacer are defined in the electron density.

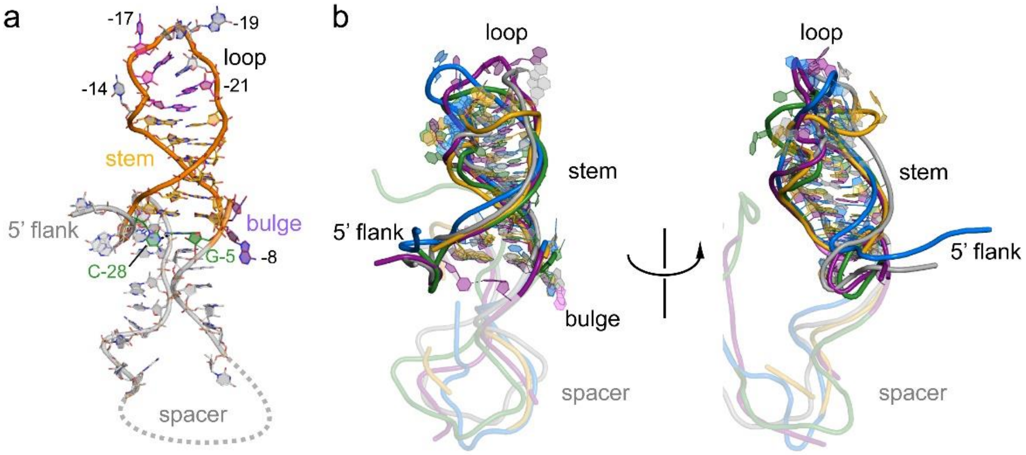

c

	5' flank (-30)	5' stem	loop (-20)	3' stem+bulge (-10)			3' flank (-1)
Rc	GCCUC|A|CAUC	ACCGCC	AAGACGAC	GGCGG	AC	U	GAACC
Lba	GCUGGAG|AAGAU	AGCCC	AAGAAAGA	GGGC	AA	U	AAC
Lbu	GAUUU A|GACC	ACCCC	AAAAAUGAA	GGGG	AC	U	AAAACA
Lsh	AUAUAGAC|C	ACCCC	AAUAUCGAA	GGGG	AC	U	AAAAC
Lse	|GACU	ACCUC	UAUAUGAAA	GAGG	AC	U	AAAACC

Supplementary Figure S11. Comparison of crRNAs of the Cas13a-family of endonucleases. a) Structure of the crRNA in the RcCas13a binary complex. The stem of the crRNA is depicted in orange, bulge and residues at the loop (grey) making base specific interactions are highlighted in violet and pink, respectively. The twisted base-pair at the bottom of the stem-loop unique to the RcCas13a bound crRNA is highlighted in green. The spacer and flanking regions are shaded in transparent grey. Disordered residues of the spacer sequence in the X-ray structure of the binary complex are indicated as dashed line. b) Superposition of crRNAs of homologous Cas13a binary complex structures. crRNAs from RcCas13a (purple, this work), LbuCas13a (grey, r.m.s.d. 1.8Å, PDB code 5XWY), LseCas13a (green, r.m.s.d. 1.2Å, PDB code 6VRC)), LbaCas13a (blue, r.m.s.d. 6.2Å, PDB code 5WLH), LshCas13a (golden, r.m.s.d 2.7Å, PDB code 5WTK). The phosphate backbone of the spacer is shown transparent. c) Sequence alignment of crRNA sequences. Shaded grey: bases forming the stem-loop; pink: bases with sequence specific contacts; blue: bases with stacking interactions with the protein; green: twisted base pair unique to the RcCas13a-bound crRNA. | indicates pre-crRNA cleavage position at the 5' flank (Labels are colored according to b).

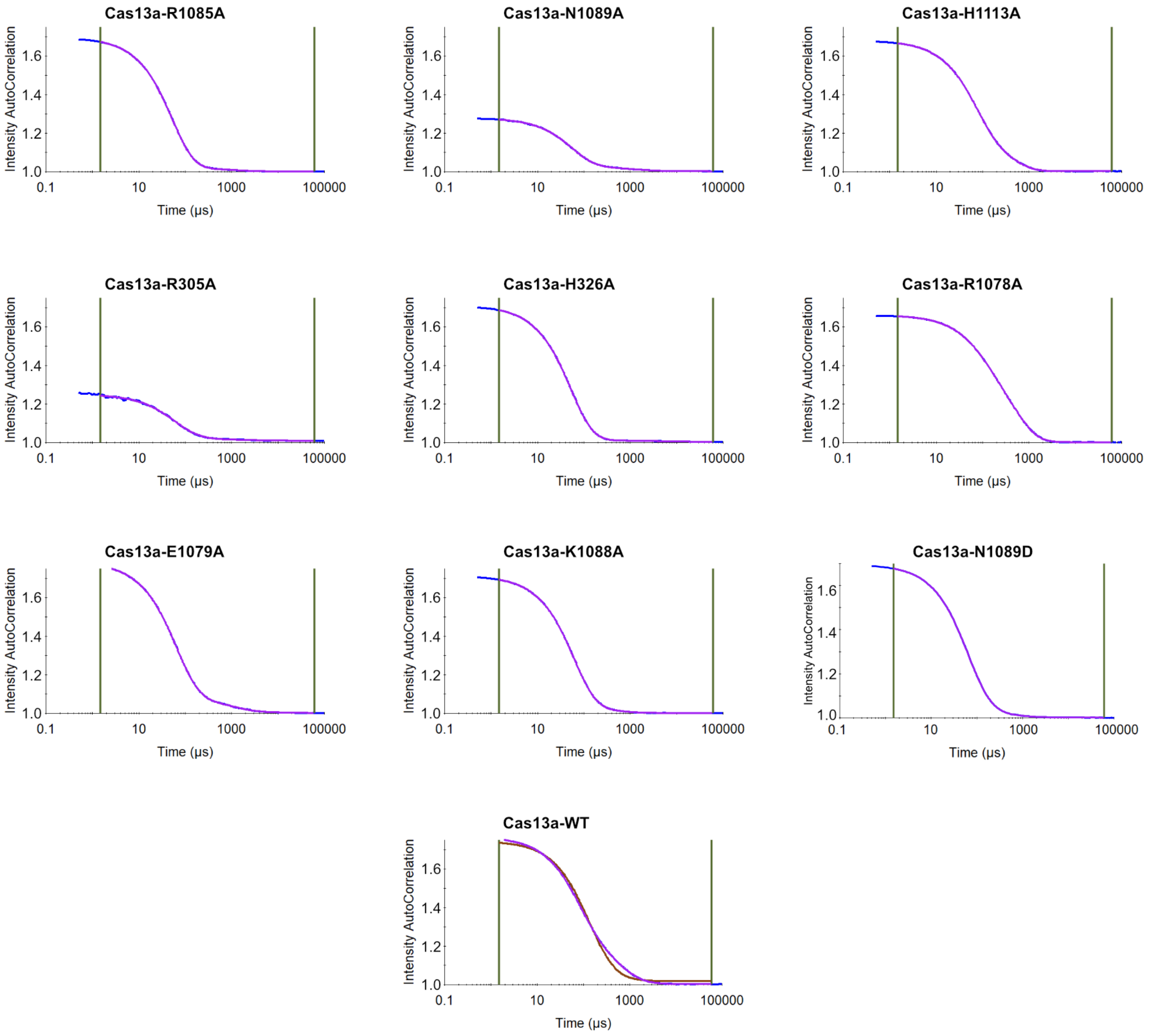

Supplementary Figure S12. Quality control of purified RcCas13a proteins by DLS-analysis, showing the integrity of all RcCas13a proteins. The observed intensity autocorrelation for the RcCas13a^{R305A} and RcCas13a^{N1089A} mutants are lower compared to the other RcCas13a proteins due to lower protein concentration.

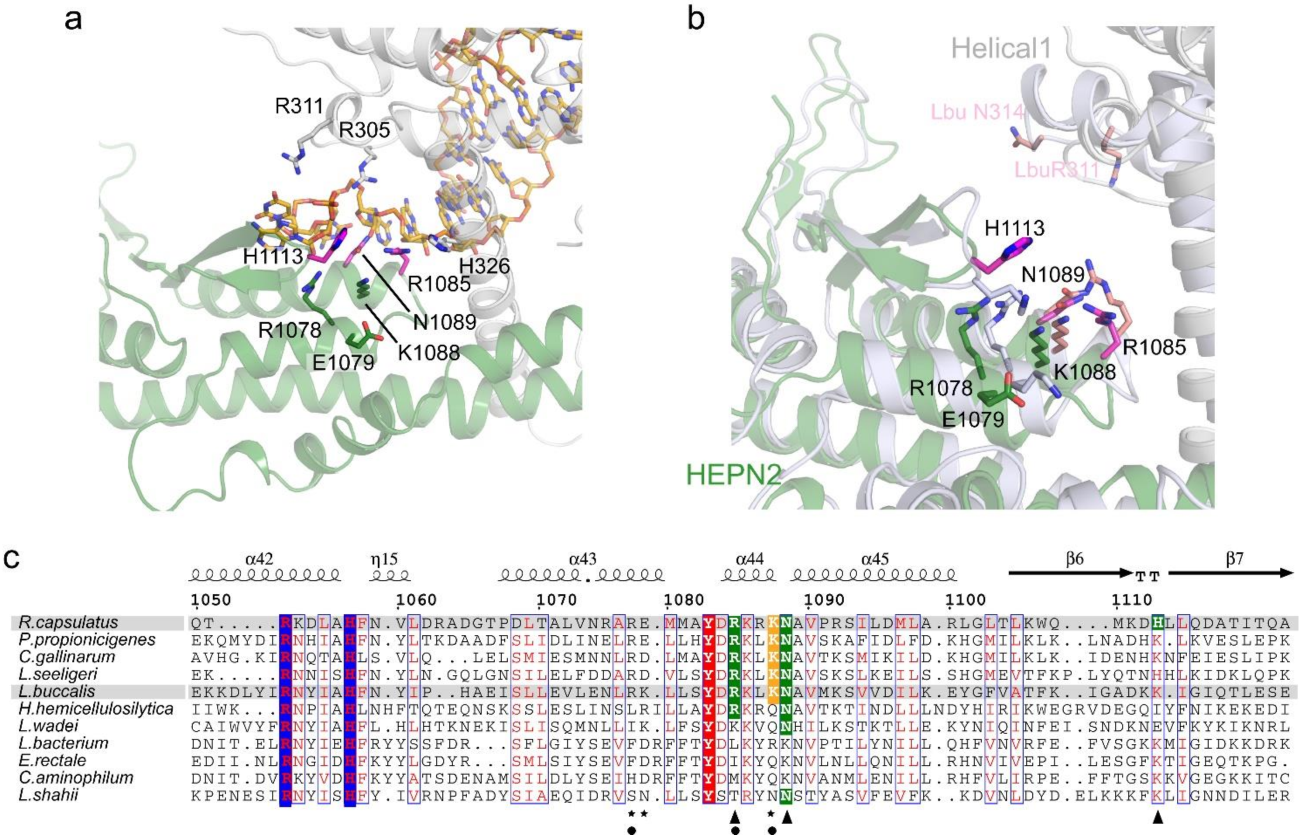

Supplementary Figure S13. Comparison of RcCas13a and LbuCas13a. a) Close-up view of the crRNA processing site in RcCas13a. Residues that were mutated to Ala are shown as stick model, with the residues important for catalysis highlighted in pink. (HEPN2= green, Hel1 = grey). b) Superposition of the binary complexes of RcCas13a and LbuCas13a (PDB code 5XWY, light blue) using the main chain atoms of the HEPN2 domain as reference frame. Residues in RcCas13a responsible for pre-crRNA processing are highlighted in pink, the corresponding residues in LbuCas13a are shown in grey. The 5' end of the crRNA in the RcCas13a binary complex is shown as orange stick model. (crRNA in the LbuCas13a binary complex is not shown for clarity). In LbuCas13a two residues (Arg 311, Asn 314) that do not make any contact with the crRNA were identified as crucial for the processing activity.(6) c) Section of a part of the sequence alignment of the HEPN2 domains, with the residues in RcCas13a involved in pre-crRNA processing highlighted in green and marked with an arrow. Residues that where mutated in RcCas13a but did not impact on the processing activity are marked with a star. The residues in LbuCas13a responsible for the pre-crRNA processing (6) are marked with a dot. Arg 1052 and His 1057 involved in target RNA cleavage are highlighted in blue.

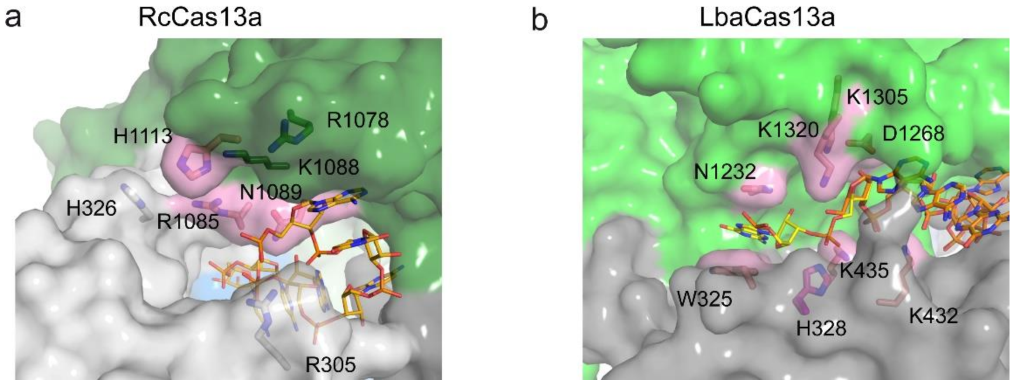

Supplementary Fig. S14. Comparison of the pre-crRNA processing site of RcCas13a and LbaCas13a. Semi-transparent surface representation of a) the RcCas13-crRNA complex and b) the LbaCas13a-H328A-pre-crRNA complex (PDB code 5WLH), superimposed with the processed crRNA and His328 side chain of the LbaCas13a-crRNA complex (PDB code 5W1H). While in the LbaCas13a complexes the 5' flank of the pre-crRNA and crRNA are tightly held by the surrounding amino acid side chains, the equivalent site in the RcCas13a complex is more open, allowing more flexibility for the 5' flank of the crRNA. Residues lining the active side are shown as stick models, with the side chains of amino acids involved in pre-crRNA cleavage highlighted in pink. The Hel1 and HEPN2 domains are colored grey and green, respectively, the crRNA and pre-crRNAs are shown as stick model. (RcaCas13a-crRNA: golden; LbaCas13a: pre-crRNA=yellow, crRNA=orange).

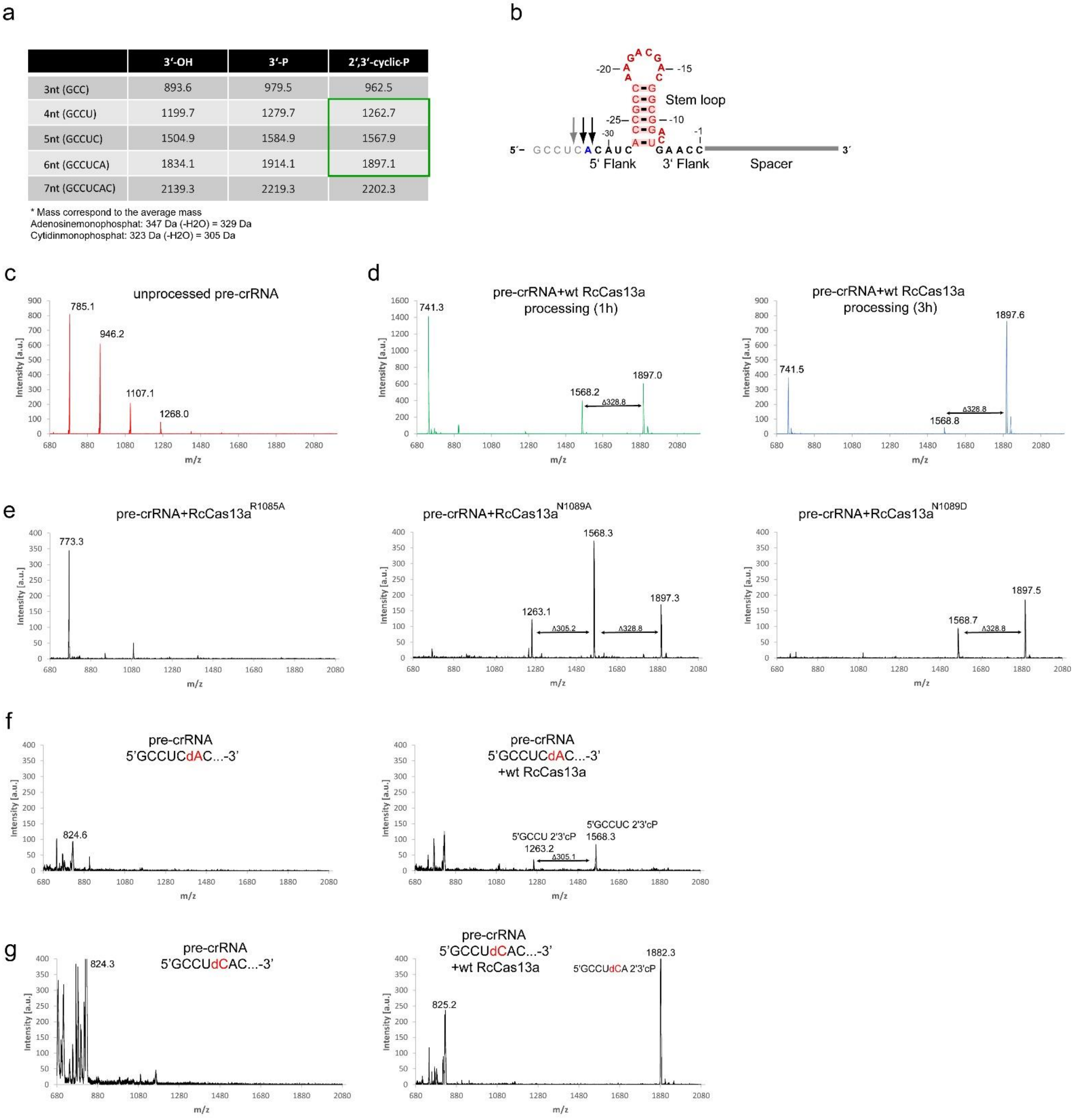

	3'-OH	3'-P	2',3'-cyclic-P
3nt (GCC)	893.6	979.5	962.5
4nt (GCCU)	1199.7	1279.7	1262.7
5nt (GCCUC)	1504.9	1584.9	1567.9
6nt (GCCUCA)	1834.1	1914.1	1897.1
7nt (GCCUCAC)	2139.3	2219.3	2202.3

* Mass correspond to the average mass
Adenosinemonophosphat: 347 Da (-H2O) = 329 Da
Cytidinmonophosphat: 323 Da (-H2O) = 305 Da

Supplementary Fig. S15. MALDI-TOF analysis of the pre-crRNA processing reaction products. a) Table with average masses of possible 5' reaction products calculated with Mongo Oligo Mass Calculator v2.06 (http://rna.rega.kuleuven.be/masspec/mongo.htm). b) Scheme of the pre-crRNA with the cleavage sites indicated by arrows. c) Spectra of pre-crRNA without incubation with RcCas13a and d) following incubation with wt RcCas13a for 1h or 3h. Masses of observed reaction products correspond to cleavage 4 or 5 bases 5' to the pre-crRNA hairpin with a 2',3' cyclic phosphate (2'3'cP). e) No pre-crRNA processing can be observed by incubation of the pre-crRNA with the inactive RcCas13a^{R1085A} mutant (left). In contrast mutation of Asn1089 to Ala leads to aberrant cleavage 6 nt's 5' to the stem loop (middle), while mutation to Asp does not impact on the cleavage position. f+g) Substitution of the adenosine by deoxyadenosine (dA) and cytosine by desoxycytosine (dC) at the observed cleavage positons 5 or 4 bases 5' to the stem loop, respectively, abrogates cleavage at this position.

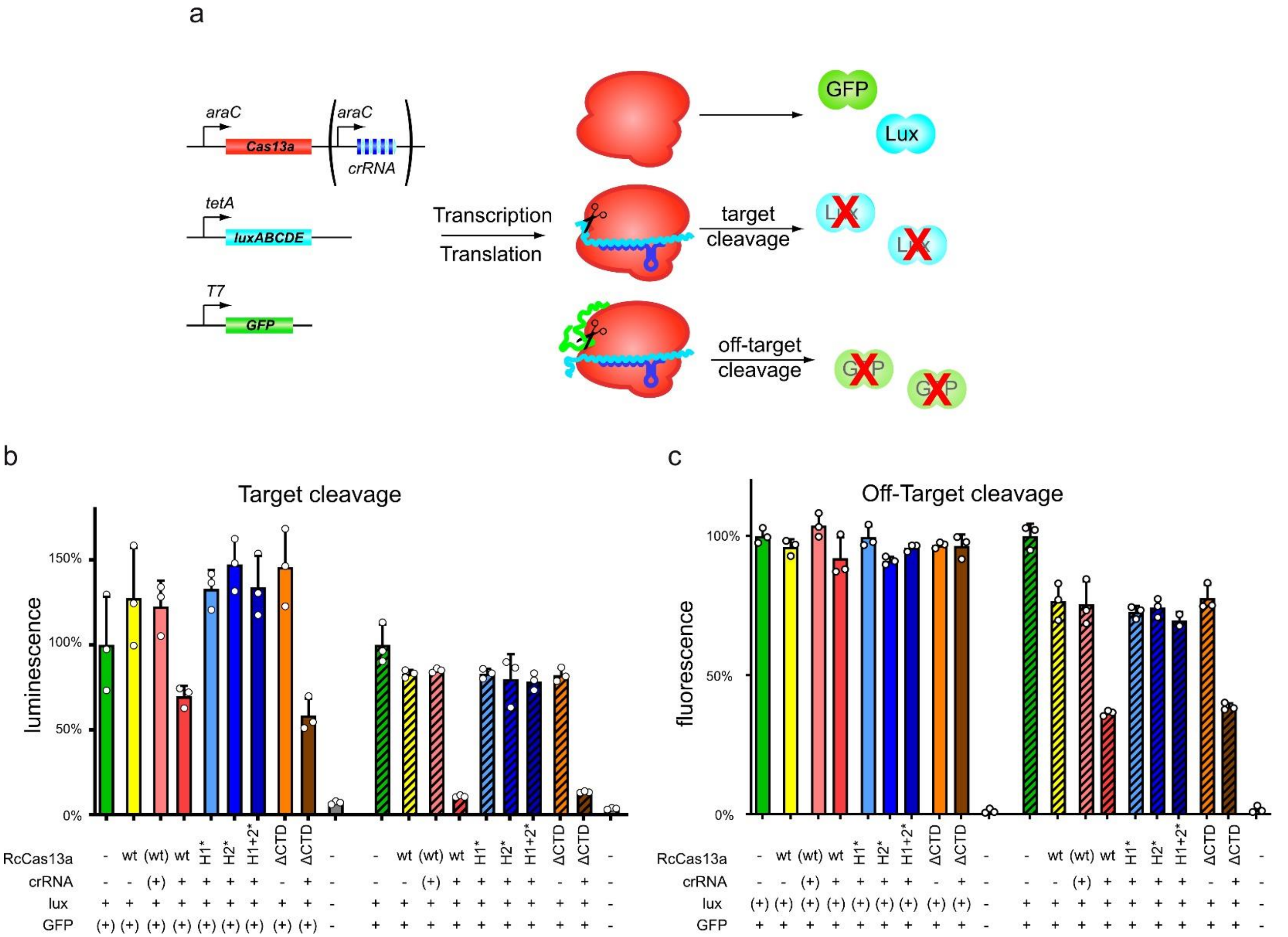

Supplementary Figure S16. *In vivo* activity of RcCas13a. a) Schematic depiction of the *in-vivo* activity assay. Two reporter gene constructs (*luxABCDE* and *GFP*) as well as expression constructs for RcCas13a variants and crRNA encoded within a CRISPR-locus targeting *luxABCDE* are introduced into *E. coli* cells. Upon expression of RcCas13a and crRNA, luminescence is inhibited by target RNA-cleavage activity of RcCas13a, while fluorescence is prevented by non-sequence specific off-target RNA-cleavage activity. b) Sequence specific target cleavage activity by RcCas13a can be followed by bioluminescence upon luciferase expression reduction. Wt RcCas13a and RcCas13aΔCTD reduce bioluminescence in a crRNA dependent manner (red and brown bars), while all HEPN-mutants lost their impact on luciferase expression (blue bars). This effect is not affected by induction of GFP expression (right, hatched bars). c) Non-sequence specific RNA cleavage was analyzed by measuring GFP-fluorescence upon induction of crRNA and target RNA expression. Wt RcCas13a and RcCas13aΔCTD reduce GFP-fluorescence only upon expression of crRNA and target sequence (red and brown bars). Mutation of any of the catalytic residues in the HEPN-domains leads to an inactive RcCas13a. (blue hatched bars). No impact on GFP-fluorescence can be observed in the absence of *luxABCDE* expression induction (left). RcCas13a and crRNA expression is induced by addition of arabinose to the growth medium. Luciferase and *GFP* expression is controlled by anhydrotetracycline and IPTG, respectively. (+): Encoding gene/expression construct is present in the *E. coli* cells, but was not induced. (-) empty vector control. (H1* = RcCas13a$^{R464A/H469A}$; H2*=RcCas13a$^{R464A/H469A}$; H1+2*=RcCas13a$^{R464A/H469A/R1052A/H1057A}$; ΔC-term = Δ C-terminal domain). Determined values for fluorescence and bioluminescence were corrected by the cell density (OD_{600}) and normalized to the empty vector control; experiments were performed in three independent replicates (n=3).

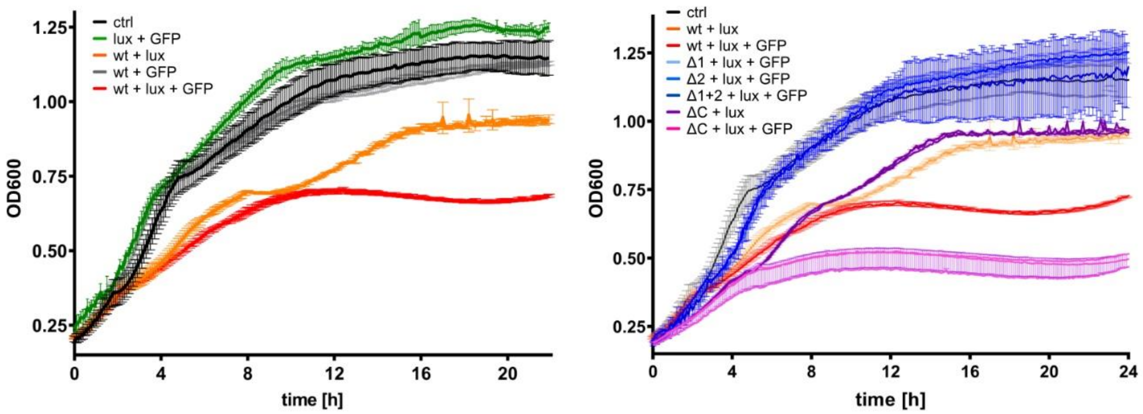

Supplementary Figure S17. Bacterial growth during *in vivo* activity. Growth curves of RcCas13a and reporter gene expressing *E. coli* JM109 (DE3) over 20 h at 30 °C. ctrl: empty vector control. Induction of expression of active RcCas13a together with crRNA and target RNA leads to a reduction in growth.

Table S1: Protein sequence of RcCas13a. The HEPN active site residues are highlighted in red, residues responsible for pre-crRNA processing in green, and the C-terminal domain (CTD) is highlighted in yellow.

RcCas13a	MQIGKVQGRTISEFGDPAGGLKRKISTDGKNRKELPAHLSSDPKALIGQWISGIDKIYRKPDSRK<br>SDGKAIHSPTPSKMQFDARDDLGEAFWKLVSEAGLAQDSDYDQFKRRLHPYGDKFQPADSGAKLK<br>FEADPPEPQAFHGRWYGAMSKRGNDAKELAAALYEHLHVDEKRIDGQPKRNPKTDKFAPGLVVAR<br>ALGIESSVLPRGMARLARNWGEEEIQTYFVVDVAASVKEVAKAAVSAAQAFDPPRQVSGRSLSPK<br>VGFALAEHLERVTGSKRCSFDPAAGPSVLALHDEVKKTYKRLCARGKNAARAFPADKTELLALMR<br>HTHENRVRNQMVRMGRVSEYRGQQAGDLAQSHYWTSAGQTEIKESEIFVRLWVGAFALAGRSMKA<br>WIDPMGKIVNTEKNDRDLTAAVNIRQVISNKEMVAEAMARRGIYFGETPELDRLGAEGNEGFVFA<br>LLRYLRGCRNQTFHLGARAGFLKEIRKELEKTRWGKAKEAEHVVLTDKTVAAIRAIIDNDAKALG<br>ARLLADLSGAFVAHYASKEHFSTLYSEIVKAVKDAPEVSSGLPRLKLLLKRADGVRGYVHGLRDT<br>RKHAFATKLPPPPAPRELDDPATKARYIALLRLYDGPFRAYASGITGTALAGPAARAKEAATALA<br>QSVNVTKAYSDVMEGRTSRLRPPNDGETLREYLSALTGETATEFRVQIGYESDSENARKQAEFIE<br>NYRRDMLAFMFEDYIRAKGFDWILKIEPGATAMTRAPVLPEPIDTRGQYEHWQAALYLVMHFVPA<br>SDVSNLLHQLRKWEALQGKYELVQDGDATDQADARREALDLVKRFRDVLVLFLKTGEARFEGRAA<br>PFDLKPFRALFANPATFDRLFMATPTTARPAEDDPEGDGASEPELRVARTLRGLRQIARYNHMAV<br>LSDLFAKHKVRDEEVARLAEIEDETQEKSQIVAAQELRTDLHDKVMKCHPKTISPEERQSYAAAI<br>KTIEEHRFLVGRVYLGDHLRLHRLMMDVIGRLIDYAGAYERDTGTFLINASKQLGAGADWAVTIA<br>GAANTDARTQTRKDLAHFNVLDRADGTPDLTALVNRAREMMAYDRKRKNAVPRSILDMLARLGLT<br>LKWQMKDHLLQDATITQAAIKHLDKVRLTVGGPAAVTEARFSQDYLQMVAAVFNGSVQNPKPRRR<br>DDGDAWHKPPKPATAQSQPDQKPPNKAPSAGSRLPPPQVGEVYEGVVVKVIDTGSLGFLAVEGVA<br>GNIGLHISRLRRIREDAIIVGRRYRFRVEIYVPPKSNTSKLNAADLVRID

Table S2: RNA sequences. FAM= 6-carboxyfluorescein; d=desoxy

**Name**	**Sequence (5' to 3' directionality)**
pre-crRNA for *in vitro* processing	GCCUCACAUCACCGCCAAGACGACGGCGGACUGAACCGGGUGUCGC<br>CCUCGAACUUCACCUCGGC-3'-FAM
dA-modified pre-crRNA for *in vitro* processing	GCCUCdACAUCACCGCCAAGACGACGGCGGACUGAACCGGGUGUCGC<br>CCUCGAACUUCACCUCGGC
dC-modified pre-crRNA for *in vitro* processing	GCCUdCACAUCACCGCCAAGACGACGGCGGACUGAACCGGGUGUCGC<br>CCUCGAACUUCACCUCGGC
crRNA for crystallization	GCCUCACAUCACCGCCAAGACGACGGCGGACUGAACCUUCAUUACC<br>UCUGUUUG
CRISPR locus targeting *luxABCDE* for *in vivo* activity assay (italic: repeat regions; underlined spacer regions with the seed regions marked as bold capital letters)	gaccugagccuca*caucaccgccaagacgacggcggacugaacc*uu<br>cauuacc**UCUGU**uugagaaaauuggggccuca*cgucaccgccaaga*<br>*cgacggcggacugaacc*cgaacuaua***CUUUG***uucuugaacaguugg<br>cauca*caucaccgccaagacgacggcggacugaacc*caccaaaau**U**<br>**AAUG**gauugcacuaaaucgccuca*caucaccgccaagacgacggcg*<br>*gacugaacc*gauccaucc**UGCGG**gcaaaaccaacgcgccuca*cauc*<br>*accgccaagaugacggcgggaacccaa*ccacacuaa**UGGAU**cgcuc<br>gaaaaaaucugcaaac
CRISPR locus targeting *luxABCDE* with scrambled spacer sequence at nt 10-14 for *in vivo* activity assay (italic: repeat regions; underlined: spacer regions with the seed regions marked as bold capital letters)	gaccugagccuca*caucaccgccaagacgacggcggacugaacc*uu<br>cauuacc**AGACA**uugagaaaauuggggccuca*cgucaccgccaaga*<br>*cgacggcggacugaacc*cgaacuaua**GAAAC**uucuugaacaguugg<br>cauca*caucaccgccaagacgacggcggacugaacc*caccaaaau**A**<br>**UUAC**gauugcacuaaaucgccuca*caucaccgccaagacgacggcg*<br>*gacugaacc*gauccaucc**ACGCC**gcaaaaccaacgcgccuca*cauc*<br>*accgccaagaugacggcgggaacccaa*ccacacuaa**ACCUA**cgcuc<br>gaaaaaaucugcaaac

Table S3: Plasmids used in this study.

Vector name	Application	Selection marker	Source / Construction
pET24-RcCas13a + crRNA	Protein expression (crystallisation)	Kanamycin	This work
p2CT-His-MBP-TEV-RcCas13a	Protein expression	Ampicillin	Addgene #91870
p2CT-His-MBP-TEV-RcCas13a*	Protein expression	Ampicillin	This work<br>*Wt and RcCas13a single mutants: R305A, R326A, R1078A, E1079A, K1088A, R1085A, N1089A, N1089D, H1113A
p2CT-RcCas13a-H1*	Protein expression	Ampicillin	This work<br>p2CT-RcCas13a$^{R464A/H469A}$
p2CT-RcCas13a-H2*	Protein expression	Ampicillin	This work<br>p2CT-RcCas13a$^{R1052A/H1057A}$
p2CT-RcCas13a-H1+2*	Protein expression	Ampicillin	This work<br>p2CT-RcCas13a$^{R464A/H469A}$<br>$^{R1052A/H1057A}$
pASK-IBA5-luxABCDE	*In vivo* activity assay	Ampicillin	This work
pACYC-eGFP-ssrA	*In vivo* activity assay	Chlor-amphenicol	This work
pBAD-RSF1031K-RcCas13a	*In vivo* activity assay	Kanamycin	This work
pBAD-RSF1031K-RcCas13a + crRNA	*In vivo* activity assay	Kanamycin	This work and CRISPR-lux-loci mutant: Scrambled
pBAD-RSF1031K-RcCas13a-H1*	*In vivo* activity assay	Kanamycin	This work<br>pBAD-RSF1031K-RcCas13a<br>R464A/H469A
pBAD-RSF1031K-RcCas13a-H2*	*In vivo* activity assay	Kanamycin	This work pBAD-RSF1031K-RcCas13a<br>$^{R1052A/H1057A}$
pBAD-RSF1031K-RcCas13a-H1+2*	*In vivo* activity assay	Kanamycin	This work<br>pBAD-RSF1031K-RcCas13a<br>$^{R464A/H469A/}$<br>$^{R1052A/H1057A}$
pBAD-RSF1031K-RcCas13a-H1* + crRNA	*In vivo* activity assay	Kanamycin	This work<br>pBAD-RSF1031K-RcCas13a + crRNA<br>R464A H469A
pBAD-RSF1031K-RcCas13a-H2* + crRNA	*In vivo* activity assay	Kanamycin	This work<br>pBAD-RSF1031K-RcCas13a + crRNA<br>R1052A H1057A
pBAD-RSF1031K-RcCas13a-H1+2* + crRNA	*In vivo* activity assay	Kanamycin	This work<br>pBAD-RSF1031K-RcCas13a + crRNA<br>R464A/H469A<br>R1052A/H1057A

Table S4: List of primers used in this study.

Internal number	Name	Sequence (5' to 3' directionality)
607	LK-22_CRISPR-loci-SalI_fw	gatcgtcgacgatccaatcgag
608	LK-23_CRISPR-loci-SbfI_rev	gatccctgcaggtcgaaaaagg
609	LK-24_C2c2-seq1_fw	tttgccgctcctcg
610	LK-25_C2c2-seq2_fw	ttccgcctgtttgc
611	LK-26_C2c2-seq3_rev	aaccaaagcggaacc
631	RcDoudna	taatacgactcactatagtcacatcaccgccaagacgacggcggactgaaccgg cttaccccatacccaagaaaggaacatcatg
632	CRISPRrev	catgatgttcctttcttgggtatg
641	LK-20_C2c2-XbaI_fw_2	gatctctagacatatgcagattggcaagg
642	LK-21-C2c2-BsaI/XhoI_rev_2	gatcctcgaggagacctcagtcgatccgcaccag
648	C2c2 3' seq fw	gtaacacgtcgaagc
649	pLIKE loci rc seq	cacgaaacaataattgg
653	LK-27_C2c2-GA_fw	caacaccaattagttaaggaggattctagacatatgcagattggcaaggttc
654	LK-28_C2c2-GA_rev	ttaggcgggctgcccggggacgtcctcgaggagacctcagtcgatccgcaccagatc
655	LK-29_loci-GA_fw	gcccctttttcatgaaactagttcgtcgacgatccaatcgagtc
656	LK-30_loci-GA_rev	cggaattagcttgcatgcctgcaggtcgaaaaaggggcagccc
665	LK-31_NcoI-blaI_fw	gatcccatggatgaaaaaaatacctcaaatctc
666	LK-32_blaI-HindIII_rev	gatcaagctttcattccttctttctgttc
667	LK-33_C2c2-TEV-correction_fw	cgagtgcggccgcaaggctctggaaatacaagttttcg
668	LK-34_C2c2-TEV-correction_rev	cgaaaacttgtatttccagagccttgcggccgcactcg
669	LK-35_C2c2-pBAD_fw	tttaagaaggagatatacatacccatggatgcagattggcaaggttc
670	LK-36_C2c2-pBAD_rev	ctgaggtacctcagtggtggtggtggtg
671	LK-37_C2c2-ara/loci_rev	tagggataggcttaccttcaagctttcagtggtggtggtggtg
672	LK-38_ara/loci-C2c2_fw	ccaccactgacggtacctcagagaagaaac
673	LK-39_ara/loci-pBAD_rev	tagggataggcttaccttcaagctttctagagtttgcagattttttc
674	LK-40_lux-pACYC_fw	accatcatcaccacagccaggatccatgaaatttggaaactttttgc
675	LK-41_lux-pACYC_rev	ctgcaggcgcgccgagctcgaattctcaactatcaaacgcttcg
681	LK-42_pBAD-rev-seq	ctctcatccgccaaaac
682	LK-43_C2c2-5'-seq-rev	gatcgcttgacagatgag
693	ACYCDuetUP1	ggatctcgacgctctccct
694	DuetDown1	gattatgcggccgtgtacaa
695	LK-44_pBAD-C2c2_fw2	tttaagaaggagatatacatacccatggatgcagattggcaaggttcaaggg
696	LK-45_C2c2-pBAD_rev2	tagggataggcttaccttcaagctttcagtgtgtgtggtggtggtg
697	LK-46_C2c2-ara/loci_rev2	ggtttcttctctgaggtaccgtcagtggtggtggtggtggtg
698	LK-47_ara/loci-C2c2_fw2	caccaccaccaccaccactgacggtacctcagagaagaaac
704	LK-48_lux-pACYC_rev2	cagcggtttctttaccagactcgagtcaactatcaaacgcttcg
703	LK-45_C2c2-pBAD_rev3	tagggataggcttaccttcaagctttcagtggtggtggtggtggtgc
705	LK-49_pBADRSF1031K_rev	ctctagaggatccccggg
706	LK-50_pBADRSF1031K_fwd	aagcttggctgttttggc
707	LK-51_C2c2_into_pBADRSF1031K_fwd	tacccggggatcctctagagatgcagattggcaaggttc
708	LK-52_C2c2_into_pBADRSF1031K_rev	ccgccaaaacagccaagcttgcttcctttcgggctttg
709	LK-53_C2c2-loci_into_pBADRSF1031K_rev	tgaggtaccggcttcctttcgggctttg

710	LK-54_ara/loci-lux_into_pBADRSF1031K_fwd	gaaaggaagccggtacctcagagaagaaac
711	LK-55_ara/loci-lux_into_pBADRSF1031K_rev	ccgccaaaacagccaagctttctagagtttgcagatttttttc
712	LK-56_pASK-rev	ccgagctcgaattcgggac
713	LK-57_pASK-fwd	cggggatccctcgaggtc
714	LK-58_luxA-E_into_pASK_fwd	ggtcccgaattcgagctcggatgaaatttggaaactttttgc
715	LK-59_luxA-E_into_pASK_rev	tcgacctcgagggatccccgtcaactatcaaacgcttcg
716	LK-60_BamHI_luxA_fwd	gatcggatccatgaaatttggaaactttttgc
717	LK-61_EcoRI_luxE_rev	gatcagaattctcaactatcaaacgcttcg
718	pASK seq rev	cgcagtagcggtaaacg
719	pBAD RSF1031 seq rev	ggtcaggtgggaccacc
730	LK-62_HindIII-loci	gatcaagcttcggtacctcagagaagaaac
731	LK-63_loci-XmnI	gatcgaagatcttctctagagtttgcagatttttttc
734	C2c2 3' colony PCR	gccagatcaaaaaccgcccaac
753	LK-64_QC-HEPN1_fw	ctgcgcggttgcgcgaaccagacctttgcccttggtgcc
754	LK-65_QC-HEPN1_rev	gcaccaagggcaaaggtctggttcgcgcaaccgcgcag
755	LK-66_QC-HEPN2_fw	cgcgcgcacccaaaccgccaaagaccttgcggctttcaatgtgc
756	LK-67_QC-HEPN2_rev	gcacattgaaagccgcaaggtctttggcggtttgggtgcgcgcgtc
764	LK-72_p2CT-C2c2-seq1	attttatcgatcccactgatcc
765	LK-73_p2CT-C2c2-seq2	attcaaacttatttcgtcgtcg
766	LK-74_p2CT-C2c2-seq3	aaggcaaaagaggcgg
767	LK-75_p2CT-C2c2-seq4	cctgaacctattgacacacg
768	LK-76_p2CT-C2c2-seq5	acgtttcttattaatgcttcaaagc
769	MBP-fw	gatgaagccctgaaagacgcgcag
770	LK-77_rrnB-T2_rev	ccatccgtcaggatggcc
771	LK-78_pET-NcoI	gatcccatgggagttggctgctgccac
772	LK-79_pET-T7-lacO-NheI	gatcgctagcggggaattgttatccgctcacaattcccctatagtgagtcgt<br>attaaagcttcctttcgggctttg
773	LK-80_loci-NcoI	gatcccatggtctagagtttgcagatttttttcg
53	pASK-Iba5for	gagttattttaccactccct
61	T7-in-vitro-TX	gatgtaatacgactcactatag
888	LK-119 QC p2CT HEPN1 fw	cgttatttacgtgggtgtgctaaccagaccttcgctctgggcgcacgtgctg
889	LK-120 QC p2CT HEPN1 rev	cagcacgtgcgcccagagcgaaggtctggttagcacacccacgtaaataacg
890	LK-121 QC p2CT HEPN2 fw	gctcgtacacaaacggctaaggaccttgcggcctttaatgtgttgg
891	LK-122 QC p2CT HEPN2 rev	ccaacacattaaaggccgcaaggtccttagccgtttgtgtacgagc
894	LK-123 p2CT-C2c2-seq6	ggattgaccccatggg
917	LK-134_ITX11-fw	ccaagtaatacgactcactatag
918	LK-135_ITX12-rev	gccgaggtgaagttc
919	LK-136_ITX13-crRNA-wt	ccaagtaatacgactcactataggcctcacatcaccgccaagacgacggcggact<br>gaaccgggtgtcgccctcgaacttcacctcggc
895	PM-1 SLIM fw1 C-term pet24	cttcaatggcagcgtccaggaaaacttgtatttccagagccttg
896	PM-2 SLIM rev1 C-term pet24	acggccgccac
897	PM-3 SLIM fw2 C-term pet24	gaaaacttgtatttccagagccttg
898	PM-4 SLIM rev2 C-term pet24	ctggacgctgccattg
899	PM-5 SLIM fw1 C-term p2CT	gctgtatttaatggaagtgtacagtaataacattggaagtggataac
900	PM-6 SLIM rev1 C-term p2CT	agctaccatctgcaag

901	PM-7 SLIM fw2 C-term p2CT	taataacattggaagtggataac
902	PM-8 SLIM rev2 C-term p2CT	ctgtacacttccattaaatacag
903	PM-9 SLIM fw3 C-term p2CT	cttccaatccaatgcaatgcaagttggtgaggtgtac
904	PM-10 SLIM rev3 C-term p2CT	tacaggttttcctcgatcc
905	PM-11 SLIM fw4 C-term p2CT	caagttggtgaggtgtac
906	PM-12 SLIM rev4 C-term p2CT	cattgcattggattggaag
926	PM-13 SLIM fw1 C-term CB453	cttcaatggcagcgtccagtaactcgagtaaggatctccag
927	PM-14	cacctcgacaaggtcagg
928	PM-15	gatgaagccctgaaagacg
956	LK-148 Cas13a-stop-Cterm fw	ggcagcgtccagtaaccaaagccacgc
957	LK-149 Cas13a-stop-Cterm rev	cgtggctttggttactggacgctgc
1006	LK-175_pACYC-fw	ggtaccctcgagtctggtaaag
1007	LK-176_pACYC-rev	atgtatatctccttcttatacttaactaatatactaagatg
1008	LK-177_eGFP-fw	tataagaaggagatatacatatggtttc
1009	LK-178_eGFP-rev	ttaccagactcgagggtac
1010	LK-179_eGFP-colony_fw	gccgaggtgaagttcg
1032	LK-187_deGFP_fw	tataagaaggagatatacatatggagcttttcactggcgttg
1033	LK-188_deGFP_rev	ttaccagactcgagggtaccttagatcccggcggcggtc
1034	LK-189_deGFP-ssrA_rev	ttaccagactcgagggtaccttaagctgccagagcgtagttttcatcat tagcagccggccggatcccggcggcggtc
1105	QC-fw-Cas13a-R1078A	ccgcgctgcggagatgatggcttac
1106	QC-rv-Cas13a-R1078A/E1079A	ttcaccaacgcggttaaatcg
1107	QC-fw-Cas13a-E1079A	ccgcgctcgtgcgatgatggctt
1106	QC-rv-Cas13a-R1078A/E1079A	ttcaccaacgcggttaaatcg
1108	QC-fw-Cas13a-K1088A	ccgtaaacgcgcgaatgctgtac
1109	QC-rv-Cas13a-K1088A	tcgtaagccatcatctcac
1110	QC-fw-Cas13a-H1113A	aatgaaagatgcgttgttgcaagacgctactattac
1111	QC-rv-Cas13a-H1113A	tgccattttaaagtcaatc
1133	QC-fw-Cas13a-R305A	tttgtgcgcggcgggtaagaatg
1134	QC-rv-Cas13a-R305A	cgcttgtaagttttctttac
1135	QC-fw-Cas13a-H326A	attgatgcgcgcgacccatgaaaacc
1136	QC-rv-Cas13a-H326A	gctaacaattccgttttg
1137	QC-fw-Cas13a-R1085A	ggcttacgacgcgaaacgcaagaatgctgtac
1138	QC-rv-Cas13a-R1085A	atcatctcacgagcgcgg
1139	QC-fw-Cas13a-N1089A	taaacgcaaggcggctgtacctcg
1140	QC-rv-Cas13a-N1089A	cggtcgtaagccatcatc
1141	QC-fw-Cas13a-N1089D	taaacgcaaggatgctgtacctc
1142	QC-rv-Cas13a-N1089D	cggtcgtaagccatcatc
1143	Rv-backbone-418	ctcgaaaaaatctgcaaactctagagaagattttcagcctgatac
1144	Fw-backbone-418	gaggtaccggcttcctttcgggctttg

Supplementary References

1. Couvin, D., Bernheim, A., Toffano-Nioche, C., Touchon, M., Michalik, J., Neron, B., Rocha, E. P. C., Vergnaud, G., Gautheret, D., and Pourcel, C. (2018) CRISPRCasFinder, an update of CRISRFinder, includes a portable version, enhanced performance and integrates search for Cas proteins. *Nucleic Acids Res.* **46**, W246-W251
2. Altschul, S. F., Gish, W., Miller, W., Myers, E. W., and Lipman, D. J. (1990) Basic local alignment search tool. *J. Mol. Biol.* **215**, 403-410
3. Larkin, M. A., Blackshields, G., Brown, N. P., Chenna, R., McGettigan, P. A., McWilliam, H., Valentin, F., Wallace, I. M., Wilm, A., Lopez, R., Thompson, J. D., Gibson, T. J., and Higgins, D. G. (2007) Clustal W and Clustal X version 2.0. *Bioinformatics* **23**, 2947-2948
4. Robert, X., and Gouet, P. (2014) Deciphering key features in protein structures with the new ENDscript server. *Nucleic Acids Res.* **42**, W320-324
5. Zimmermann, L., Stephens, A., Nam, S. Z., Rau, D., Kubler, J., Lozajic, M., Gabler, F., Soding, J., Lupas, A. N., and Alva, V. (2018) A completely reimplemented MPI Bioinformatics Toolkit with a new HHpred server at its Core. *J. Mol. Biol.* **430**, 2237-2243
6. Knott, G. J., East-Seletsky, A., Cofsky, J. C., Holton, J. M., Charles, E., O'Connell, M. R., and Doudna, J. A. (2017) Guide-bound structures of an RNA-targeting A-cleaving CRISPR-Cas13a enzyme. *Nat. Struct. Mol. Biol.* **24**, 825-833
