# Supplementary material for: Structure and mechanism of the RNA dependent RNase Cas13a from Rhodobacter capsulatus
Source: Commun Biol. 2022 Jan 20;5:71. doi: 10.1038/s42003-022-03025-4 (PMC8776769; doi:10.1038/s42003-022-03025-4)
Supplement: Supplementary file 3 — Description of Additional Supplementary Files [file 42003_2022_3025_MOESM3_ESM.pdf]

**Description of Additional Supplementary Files**

**File name:** Supplementary Data 1

**File type:** Microsoft Excel

**Fig. 4b and 4c:** To obtain relative fluorescence and relative luminescence, the fluorescence and luminescence values were normalized to the respective OD600 obtained at the time point 20 h. The results were set in relation to each other as a percentage. For experimental reproducibility of in vivo experiments, standard error of the mean was calculated using three independent biological experiments (n=3). For visualization a scatter plot with bar (mean with SD) was chosen. The error bars were calculated as standard deviation of three independent experiments.

- **Fig. 4b:** First bar graph: 100 % corresponds to the sample lux1 (termed bar 1 in the raw data sheet). Second bar graph: 100 % corresponds to the sample lux2 (termed bar 11 in the raw data sheet)
- **Fig. 4c:** First bar graph: 100 % corresponds to the sample (termed bar 1 in the raw data sheet)

**OD600:** Data points of OD600, luminescence and fluorescence measurements (3 worksheets within the Excel file).
